# Untargeted Metabolomics Profiling Reveals Perturbations in Arginine-NO Metabolism in Middle Eastern Patients with Coronary Heart Disease

**DOI:** 10.3390/metabo12060517

**Published:** 2022-06-03

**Authors:** Ehsan Ullah, Ayman El-Menyar, Khalid Kunji, Reem Elsousy, Haira R. B. Mokhtar, Eiman Ahmad, Maryam Al-Nesf, Alka Beotra, Mohammed Al-Maadheed, Vidya Mohamed-Ali, Mohamad Saad, Jassim Al Suwaidi

**Affiliations:** 1 Qatar Computing Research Institute, Hamad Bin Khalifa University, Doha P.O. Box 5825, Qatar; eullah@hbku.edu.qa (E.U.); kkunji@hbku.edu.qa (K.K.); msaad@hbku.edu.qa (M.S.); 2 Clinical Research, Trauma & Vascular Surgery, Hamad Medical Corporation, Doha P.O. Box 3050, Qatar; aelmenyar@hamad.qa; 3 Department of Clinical Medicine, Weill Cornell Medical College, Doha P.O. Box 24144, Qatar; 4 Department of Cardiology, Heart Hospital, Hamad Medical Corporation, Doha P.O. Box 3050, Qatar; reemelsousy@gmail.com; 5 Anti-doping Lab Qatar, Doha P.O. Box 27775, Qatar; hmokhtar@adlqatar.qa (H.R.B.M.); eahmad@adlqatar.qa (E.A.); abeotra@adlqatar.qa (A.B.); mohammed.almaadheed@adlqatar.qa (M.A.-M.); vali@adlqatar.qa (V.M.-A.); 6 Department of Internal Medicine, Allergy and Immunology, Hamad General Hospital, Hamad Medical Corporation, Doha P.O. Box 3050, Qatar; mariamali@hamad.qa

**Keywords:** metabolomics, coronary heart disease, arginine metabolism, metabolite risk score, Middle East

## Abstract

Coronary heart disease (CHD) is a major cause of death in Middle Eastern (ME) populations, with current studies of the metabolic fingerprints of CHD lacking in diversity. Identification of specific biomarkers to uncover potential mechanisms for developing predictive models and targeted therapies for CHD is urgently needed for the least-studied ME populations. A case-control study was carried out in a cohort of 1001 CHD patients and 2999 controls. Untargeted metabolomics was used, generating 1159 metabolites. Univariate and pathway enrichment analyses were performed to understand functional changes in CHD. A metabolite risk score (MRS) was developed to assess the predictive performance of CHD using multivariate analysis and machine learning. A total of 511 metabolites were significantly different between the CHD patients and the controls (FDR *p* < 0.05). The enriched pathways (FDR *p* < 10^−300^) included D-arginine and D-ornithine metabolism, glycolysis, oxidation and degradation of branched chain fatty acids, and sphingolipid metabolism. MRS showed good discriminative power between the CHD cases and the controls (AUC = 0.99). In this first study in the Middle East, known and novel circulating metabolites and metabolic pathways associated with CHD were identified. A small panel of metabolites can efficiently discriminate CHD cases and controls and therefore can be used as a diagnostic/predictive tool.

## 1. Introduction

Coronary heart disease (CHD) is a major cause of mortality worldwide [1]. Many risk factors have been identified that contribute to CHD, including genetic, lifestyle, and environmental mediators [2]. Greater insight into biological processes of CHD is needed to better understand and improve its diagnosis and treatment. The advent of omics technology such as genomics, transcriptomics, and metabolomics has significantly aided the discovery of novel risk markers, as well as the elucidation of mechanisms related to the etiology and pathogenesis of CHD. Metabolomics is a type of omics data that is understudied for CHD and could provide diagnostic, prognostic, and therapeutic insights [3].

Changes in metabolism could both lead to CHD and be a consequence of the disease itself [4]. At the level of the heart, alterations in cardiac metabolism may have a direct impact on the contractile function [5]. These alterations impact the uptake of the substrates used for biological functions, such as cell growth and regeneration, resulting in different pathologies [6]. Under normal conditions, 60–90% of the energy used by the heart is generated by mitochondrial oxidation of fatty acids, and the remaining is supplied by glucose, lactate, and ketones [6].

CHD patients usually have comorbidities that accelerate the disease progression. Many of these are diseases of metabolic dysfunction, such as diabetes mellitus, dyslipidemia, and obesity, which impact many body organs and tissues, including the liver, skeletal muscle, vasculature, and myocardium [7,8,9]. These comorbidities influence the trajectory of the onset of CHD, but CHD can occur in their absence as well [9].

Previous studies have identified novel CHD risk factors using metabolomic data, but this has mainly been in individuals of European ancestries [10,11,12], and more recently in individuals of African ancestries [13]. CHD is rampant in the Middle East (ME) and Gulf region, especially in young individuals [14]. Metabolomics for CHD has not as yet been studied in ME populations. In this study, we investigated changes in systemic metabolites in CHD using untargeted metabolomics in a large ME cohort collected by the Qatar Cardiovascular Biorepository (QCbio) [14] and the Qatar Biobank (QBB) [15]. We investigated several research questions including: (1) Are there differences in metabolite levels between CHD patients and controls; (2) are there changes in metabolite groups (pathways) that are not by chance and that indicate systematic alterations in the activity level of the pathways; and (3) can multivariate analysis and machine learning models be developed as a metabolite risk score (MRS) to assess predictive performance of CHD using metabolic profiles?

## 2. Results

### 2.1. Univariate Analysis

**Figure 1:**
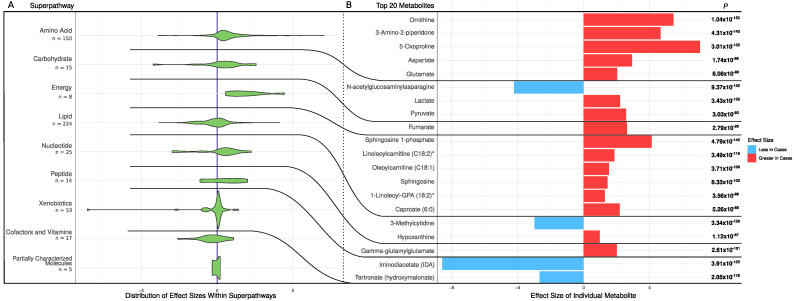
Metabolic differences between CHD patients and controls. (**A**) Distribution of effect sizes of metabolites grouped by super pathways. (**B**) Effect sizes of top 20 significantly different metabolites in cases and controls. *p* and effect size of the metabolites were calculated using a logistic regression adjusting for age, sex, and BMI.

A total of 511 metabolites were significantly different between the CHD patients and the controls (FDR *p* < 0.05). Most metabolites (331 out of 511, 65%) were elevated in the CHD cases, including amino acids, carbohydrates, energy-related metabolites, nucleotides, peptides, and xenobiotics (Figure 1A). The 20 most significantly altered metabolites had FDR *p* < 5.26 × 10^−86^ and are shown in Figure 1B. Of these, there were five amino acid metabolites (all elevated in CHD), three carbohydrates metabolites (two elevated in CHD), one energy metabolite (elevated in CHD), six lipid metabolites (all elevated in CHD), two nucleotide metabolites (one elevated in CHD), one peptide metabolite (elevated in CHD), and two xenobiotic metabolites (both decreased in CHD) (Figure 1B).

### 2.2. Pathway Enrichment Analysis

The results of the metabolic pathway enrichment analysis are shown in Table 1 for the 45 most significant pathways with FDR *p* < 10^−300^. The enriched pathways included D-arginine and D-ornithine metabolism (enrichment ratio (ER) = 760.0); glycolysis (ER = 572.2); arginine and proline metabolism (ER = 441.3); oxidation of branched chain fatty acids (ER = 429.3); sphingolipid metabolism (ER = 341.8); and valine, leucine, and isoleucine degradation (ER = 328.1). In the following sections, pathways of relevance in CHD have been highlighted.

### 2.3. Alternation in Arginine and Ornithine Metabolism and Synthesis

**Figure 2:**
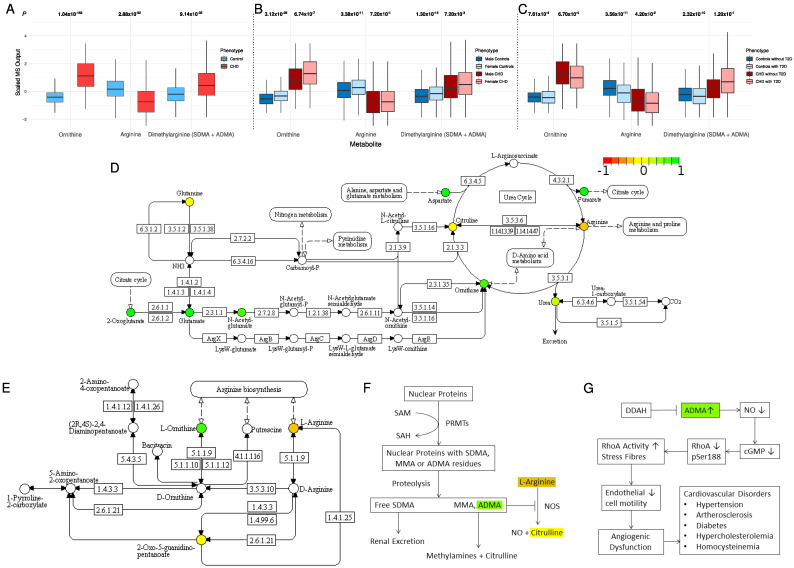
(**A**) The distribution of ornithine, arginine, and dimethylarginine in CHD patients and controls. (**B**) The impact of sex on ornithine, arginine, and dimethylarginine distribution. (**C**) The impact of type 2 diabetes on ornithine, arginine, and dimethylarginine distribution. (**D**) The arginine biosynthesis pathway (KEGG pathway: hsa00220). (**E**) The arginine and ornithine metabolism pathway (KEGG pathway: hsa00472). (**F**) The role of increased concentrations of ADMA in the metabolism of L-arginine. (**G**) The DDAH/ADMA pathway and the role of increased concentrations of ADMA in vascular function and homeostasis. Colored metabolites were found in our metabolomic profile. Metabolites in red, yellow, and green have lower, the same, and higher concentration in cases compared with controls, respectively.

In the D-arginine and D-ornithine metabolism pathway (the second most-enriched pathway; Table 1), we observed a lower concentration of arginine in CHD (FDR *p* = 2.88 × 10^−82^) and higher concentrations of both ornithine (*p* = 1.04 × 10^−152^) and total dimethylarginine (symmetric and asymmetric, i.e., SDMA, ADMA; *p* = 9.14 × 10^−35^) (Figure 2A). L-arginine is metabolized by two competing pathways (Figure 2D–G). In the first pathway, nitric oxide synthase (NOS) converts L-arginine to nitric oxide (NO) and citrulline. This is catalyzed by endothelial nitric oxide synthase (eNOS) (EC 1.14.13.39). In the second pathway, L-arginine is the substrate of arginase (EC 3.5.3.10) that produces ornithine and urea. Within cases, the metabolite concentration of ornithine was approximately twice the concentration of arginine (ornithine-to-arginine ratio (OAR) = mean concentration of ornithine divided by mean concentration of arginine = 1.94). Within the controls, however, an opposite trend was observed (OAR = 0.89), indicating a shift in arginine metabolism for higher production of ornithine. The reduced production of NO may be attributed in part to the higher inhibitory effect of ADMA (in CHD patients) on nitric oxide synthase (Figure 2F,G).

Stratifying the analysis by sex, a gender difference was observed for ornithine concentration in both the CHD and the control groups (*p* = 3.12 × 10^−28^ and 6.74 × 10^−7^, respectively; Figure 2B). Arginine concentration was similar between females and males in the CHD group (*p* = 0.72), but female controls had higher concentrations of arginine than male controls (*p* = 3.38 × 10^−11^). Females in both the CHD and the control groups had higher concentrations of dimethylarginine than males (*p* = 0.0072 and 1.3 × 10^−14^, respectively; Figure 2B). The OAR was 2.01 in CHD males and 1.83 in CHD females. These ratios indicate a shift in arginine metabolism for higher production of ornithine in male patients compared with female patients.

Stratifying the analysis by type 2 diabetes (T2D), we observed that concentrations of arginine and ornithine were significantly lower in subjects with T2D compared with subjects without T2D (Figure 2C). This indicates less availability of arginine to produce NO, which is a key component for vascular function as well as homeostasis. Interestingly, the concentrations of dimethylarginine were higher in the CHD patients who had T2D than in the CHD patients without T2D, although the difference was not statistically significant (*p* = 0.12), most likely because of the high data variability and small sample size. The opposite trend was observed in the controls at statistical significance (*p* = 2.32 × 10^−10^). This supports the hypothesis of the lower production of NO in CHD patients with T2D by inhibiting nitric oxide synthase. The OAR was 0.88 in controls without T2D, 0.98 in controls with T2D, 1.96 in CHD cases without T2D, and 1.92 in CHD cases with T2D (Figure 2C). These ratios indicate a higher production of ornithine in controls with T2D compared with the controls who do not have T2D.

### 2.4. Changes in Acylcarnitines, Branched Chain Amino Acids, Sphingolipids, and Sugar Metabolisms

**Figure 3:**
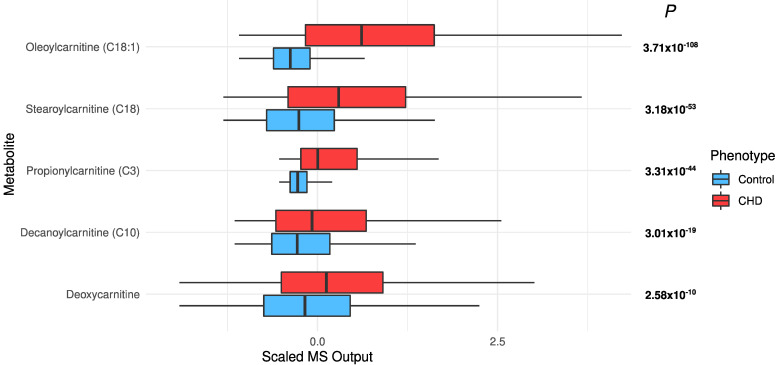
The distribution of significant acylcarnitines in the CHD patients and the controls.

**Figure 4:**
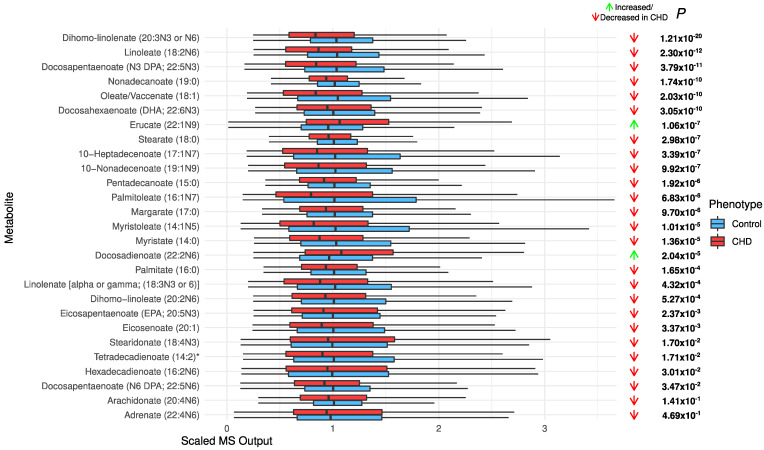
The distribution of long-chain chain fatty acids in the CHD patients and the controls.

*Increased acylcarnitines and decreased long-chain fatty acids in CHD*: All significant acylcarnitines were elevated in the CHD patients: oleoylcarnitine (C18:1) (FDR *p* = 3.71 × 10^−108^), stearoylcarnitine (C18) (FDR *p* = 3.18 × 10^−53^), propionylcarnitine (C3) (FDR *p* = 3.31 × 10^−44^), decanoylcarnitine (10) (FDR *p* = 3.01 × 10^−19^), and deoxycarnitine (FDR *p* = 2.58 × 10^−10^) (Figure 3). The most significant long-chain fatty acids (23 out of 25) were decreased in CHD (Figure 4). This may be attributed to their conversion to acylcarnitines, which were increased in CHD.

**Figure 5:**
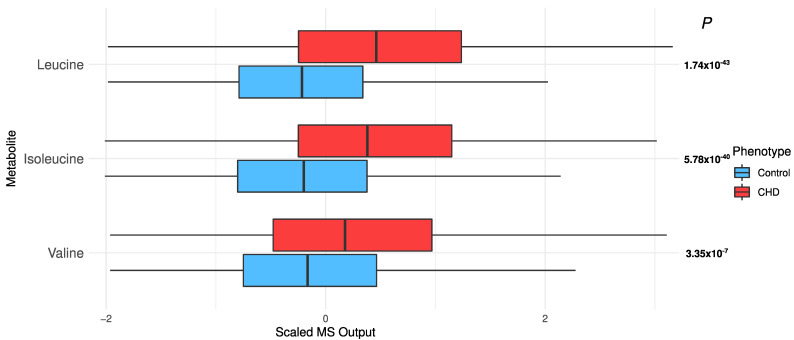
The distribution of branched chain amino acids in the CHD patients and the controls.

*Elevated branched chain amino acids (BCAAs)*: BCAAs, i.e., leucine, isoleucine, and valine, were significantly elevated in the CHD patients (leucine, *p* = 1.74 × 10^−43^; isoleucine, *p* = 5.78 × 10^−40^; and valine, *p* = 3.35 × 10^−7^; Figure 5).

**Figure 6:**
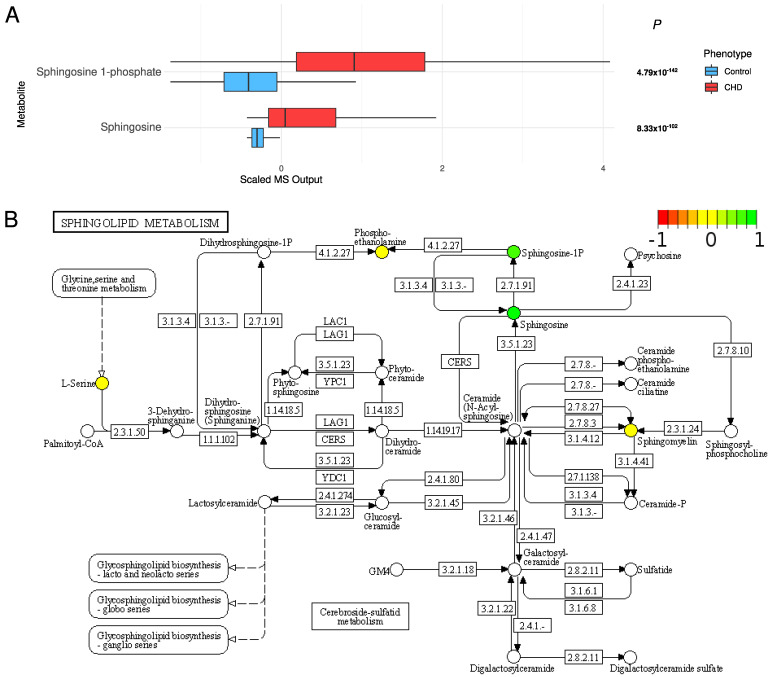
(**A**) The distribution of sphingolipids in the CHD patients and the controls. (**B**) The sphingolipid metabolism pathway (KEGG pathway: hsa00600). Colored metabolites are found in our metabolomic profile. Metabolites in red, yellow, and green have lower, the same, and higher concentrations compared with controls, respectively.

*High concentrations of sphingolipids*: Sphingolipids were significantly increased in the CHD patients: sphingosine 1-phosphate (S1P) (FDR *p* = 4.79 × 10^−142^) and sphingosine (FDR *p* = 8.33 × 10^−102^) (Figure 6).

**Figure 7:**
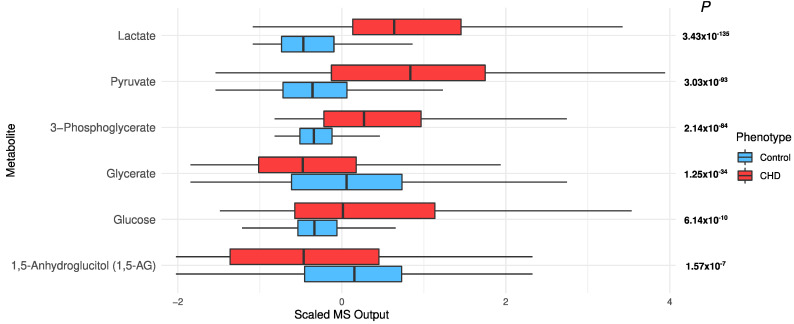
The distribution of sugars in the CHD patients and the controls.

*Sugar metabolism*: All six “glycolysis, gluconeogenesis, and pyruvate metabolism” metabolites were significantly different, with FDR *p* ≤ 8.25 × 10^−8^ (Figure 7). Lactate (*p* = 3.43 × 10^−135^), pyruvate (*p* = 3.03 × 10^−93^), and glucose (*p* = 6.14 × 10^−10^) were higher in cases than controls (Figure 7). After adjusting for T2D, age, sex, and BMI in the univariate model, glucose and 1,5-anhydroglucitol (1,5-AG) were not significant (*p* = 0.35 and 0.5, respectively), but the remaining metabolites remained very significant (*p* ≤ 3.77 × 10^−32^; data not shown).

### 2.5. Multivariate Analysis and Predictive Model

**Figure 8:**
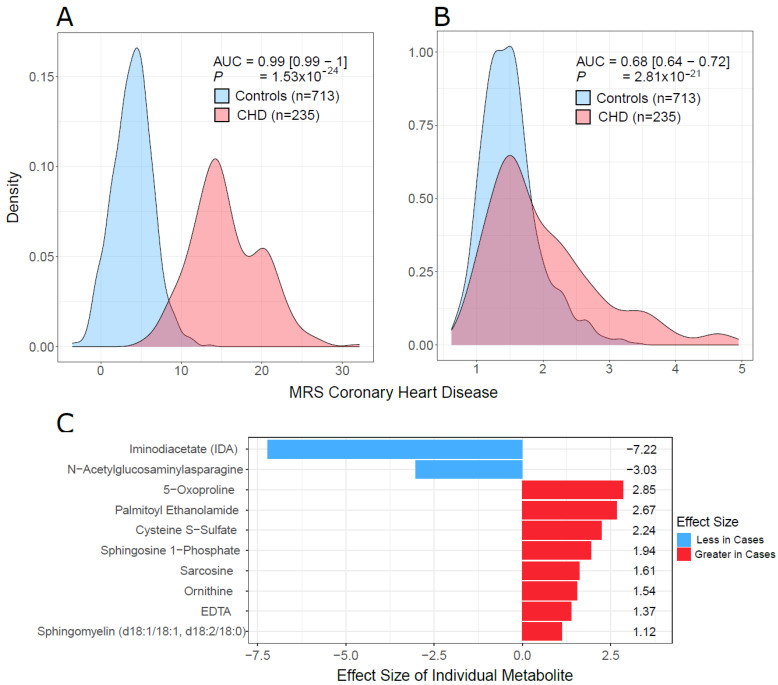
MRS discriminative performance. (**A**) Density plots of MRS10 in cases and controls in our test data. (**B**) Density plots of MRS19, which was developed by Wang et al. [13] on our test data. (**C**) The list of 10 metabolites used to build MRS10 using LASSO and their effect sizes. *p* (in (**A**,**B**)) was the *p* value from the logistic regression: CHD Status ~ MRS+ age + sex + BMI. AUC = area under the receiver operating curve, was calculated for the model that only contained the MRS (omitting all covariates) because of unbalance of cases and controls with respect to covariates.

The random forest (RF) models on the test set yielded area under the receiver operator curve (AUC) values of 0.97 and 0.98 using the full model, i.e., A (641 metabolites), and the reduced model, i.e., B (17 metabolites), respectively. The accuracies of models A and B were 98.5% and 98.7%, respectively. The mean decrease in accuracy (variable importance) of the random forest model trained on all metabolites is shown in Supplementary Figure S2. The AUCs for males and females were 1.0 and 0.97, respectively, indicating that the performance of the RF model is consistent for females and males (data not shown). Using the LASSO approach on the training data, we built a metabolite risk score using the 107 metabolites with non-zero coefficients (MRS107) (Supplementary Figure S3). MRS107 discriminated CHD patients and controls in the test data with AUC of 0.998. A reduced MRS, MRS10, was also built on 10 metabolites with the highest LASSO coefficients selected based on the knee point (Supplementary Figure S3). MRS10 performance was similar to that of MRS107, and the AUC did not decrease much (AUC = 0.99, 95% CI [0.99–1], *p* = 1.53 × 10^−24^; Figure 8A, Supplementary Figure S3). We tested the model developed by Wang et al. [13] on our test data. The performance was worse, and the AUC was much smaller (AUC = 0.68, 95% CI [0.64–0.72], *p* = 2.81 × 10^−21^; Figure 8B). The metabolites in MRS10 and their LASSO effect sizes are shown in Figure 8C. We investigated the predictive performance of MRS10 for samples with T2D by removing all the subjects in the test set who did not have T2D. AUC was 0.99, indicating that the predictive performance of our model on T2D is as good as its performance on the overall dataset.

## 3. Discussion

This is the first study that has investigated the metabolic profile and its impact on CHD in a large case-control cohort from the Middle East, using an untargeted metabolomics approach. Our results provide strong evidence that many circulating metabolites and metabolic pathways are altered in CHD patients. The results also showed that a small panel of metabolites can efficiently discriminate CHD cases and controls and can therefore be used as a diagnostic/predictive tool. In this study, we confirm known metabolites that are associated with CHD, identify novel ones, and provide insights into some underlying biological mechanisms for CHD.

Ornithine and arginine were significantly associated with CHD. The ornithine concentration in the CHD patients was twice the concentration of arginine, unlike in the controls, in whom arginine was higher. As arginine is the only physiological substrate for NOS-mediated generation of NO [16,17], our results indicate a reduced bioavailability of NO in CHD patients. NOS and arginase compete for a common substrate, L-arginine. As NOS is the only known producer of NO [17], competition for its substrate through increased arginase activity is likely to lead NOS being substrate starved. This has been observed in other non-metabolomics studies [18,19,20,21]. A higher concentration of the NOS antagonist ADMA also contributes to inhibition of NO production. Moreover, increased activity of arginase is also found in diabetes [22,23], and inhibition of arginase is found to improve endothelial function [24,25]. Diminished NO bioactivity may cause constriction of coronary arteries during exercise or during mental stress and could contribute to the provocation of myocardial ischemia in patients with CHD [26]. Thus, it could be a potential target for cardiovascular treatment. In addition, our results showed a lower concentration of arginine in T2D subjects, both in controls and in CHD patients. Male controls showed lower levels of arginine than female controls, which may be associated with the lower CHD prevalence in females.

Our analysis has revealed increased concentrations of acylcarnitines, which is associated with many heart problems such as CHD [11,27,28]. Long-chain fatty acids are converted to acylcarnitines in mitochondria via beta-oxidation. This conversion allows long-chain fatty acids to be transported across the mitochondrial membrane [29]. The increased transport can lead to storage of excess triglycerides in the cell. The generation of toxic triglyceride intermediates can lead to cellular and organ dysfunction. Moreover, elevation of long-chain acylcarnitines is associated with incomplete oxidation of fatty acids and results in insulin resistance [30,31].

BCAAs were higher in CHD patients as well. These are the main source of nitrogen for production of glutamine and alanine in muscles. In myocardial ischemia, an important oxidative energy substrate of the heart may be BCAAs, which are produced by muscle protein mobilization [32]. Accretion of BCAAs and catabolic products is attributed to heart diseases [33] such as heart failure, supporting our findings. One of the potential mechanisms of BCAAs and cardiac dysfunction is through myocardial mTOR signaling [34]. Catabolic flux modulation of BCAAs has been proposed as a potential therapy for heart failure [33,35].

Our CHD patients were found to have higher concentrations of sphingolipids than the controls. Sphingolipids were recently identified as cholesterol-independent biomarkers of CHD [36], and changes in sphingolipid metabolism, distribution, signaling, and concentration have been observed in cardiovascular diseases [37]. Sphingolipids have been previously shown to be increased in CHD [38]. Sphingolipids are active components of cell membranes and perform intracellular signal transduction and regulation of other cellular processes [38]. They are known to support essential functions in cardiogenesis and cardiac function and to mediate pathological processes [39].

Sugar metabolism was also increased in the CHD patients. Cardiac contractility requires a constant supply of adenosine triphosphate (ATP). Under normal conditions, the high demand of ATP is primarily satisfied by fatty acid oxidation (FAO), with a small contribution from glucose metabolism [7]. Under stress conditions as well as in cardiovascular diseases, there is a shift in the supply of ATP from FAO to glucose utilization [40]. Conditional analysis on T2D showed that most of the sugar metabolites remained associated with CHD, independent of T2D.

Efforts have been made to develop clinical data-based predictive models, but the accuracy of these models in CHD is <75% [41]. We have developed a predictive model to classify a metabolomic profile for CHD in this cohort. Our results were compared to those reported by Wang et al. [13], who developed a model based on metabolomics data. We developed several models to explore different aspects of predictive modeling in metabolomics. A good model can be built by using all the metabolites, but such a model is not practically feasible in a clinical setting as it will require too many metabolites for prediction. We used variable importance to select a small subset of metabolites that can generate a model with good predictive performance. The model we developed from our data had better predictive performance for our ME population than did Wang et al.’s [13] model. Wang et al.’s MRS19 had worse performance than the LASSO MRS10 developed in our cohort. This may be due to the differences in ancestries between studies, which stresses the need to study diverse populations.

## 4. Materials and Methods

### 4.1. Study Population

The study cohort comprised 1001 CHD patients and 2999 control subjects (Table 2). The CHD patients were recruited as part of QCbio, a prospective study to establish a biorepository of plasma and DNA from Qatari patients with CHD [14]. The patients were recruited between October 2013 and February 2018. The CHD patients were identified from the Cardiac Catheterization Laboratory, Coronary Care Unit, and Heart Hospital Clinics at Hamad Medical Corporation, Doha, Qatar. Patients with a history of acute coronary syndrome or stable angina were included in the study. The control subjects were recruited by the Qatar Biobank [15]. All participants were enrolled in the study after giving written informed consent. The informed consent document conformed to the guidelines regarding bioethics resources and human subject research and International Society of Biological and Environmental Biorepositories. Females and males were approximately equally represented in the control cohort, but the CHD cohort included more females (62%). The CHD patients were older than the controls (mean ± SD years = 52.7 ± 14.5 for cases and 39.8 ± 12.0 for controls; *p* < 2.2 × 10^−16^; Table 2).

### 4.2. Metabolomic Profiling and Quality Control

Serum metabolites for both the cases and the controls were jointly quantified by untargeted, ultrahigh-performance liquid chromatography-tandem mass spectroscopy (UPLC-MS/MS) and curated by Metabolon Inc. Samples were run in analytical plates containing 144 experimental samples per plate (batch). The data were normalized across batches to generate batch-normalized data and to correct for minor instrument technical variation from batch to batch. Each compound was corrected in instrument batch blocks by registering the medians of each batch to equal one and normalizing each data point proportionally. A total of 1159 metabolites were profiled within nine super pathways: lipid (30.1%), xenobiotics (21.1%), amino acid (17.3%), nucleotide (3.1%), peptide (2.9%), cofactors and vitamins (2.6%), carbohydrate (1.9%), partially characterized molecules (1.1%), energy (0.7%), and unnamed metabolites (19.2%). Unnamed metabolites were removed, leaving 937 metabolites for analysis.

Quality control steps were conducted to ensure data quality. Samples and metabolites with more than 80% missing data were removed based on the criteria suggested by Wei et al. [42]. This led to the exclusion of 7 samples and 296 metabolites. Missing values in the remaining data were imputed by replacing the missing values for each metabolite with the minimum value detected for the metabolite. Sample outliers were identified using principal component analysis (PCA) if the first five principal component values fell outside [µ ± 5 SD] (40 outliers removed; Supplementary Figure S1). Metabolites in the remaining samples were winsorized using 80% winsorization: Values for a metabolite below the 10th percentile were set to the 10th percentile, and values above the 90th percentile were set to the 90th percentile. A total of 641 metabolites among 3953 samples remained for subsequent analysis.

### 4.3. Univariate Statistical Analysis

Individual metabolite differences between the CHD patients and the controls were tested using logistic regression in R (R Core Team, version 3.6, Vienna, Austria; https://www.R-project.org/) adjusting for age, sex, and BMI to mitigate the correlations between metabolites and age, sex, and BMI. FDR corrected *p*-values (*p*) from the models were used to identify significantly different metabolites between the CHD cases and the controls, and the effect size was used to identify the direction of the changes in the metabolite concentrations with respect to disease status. A metabolite has a positive effect size if its concentration is higher in CHD patients compared with controls.

### 4.4. Pathway Enrichment Analysis

Pathway enrichment analysis provides mechanistic insight into gene lists generated from genome-scale (omics) experiments. This method identifies biological pathways that are more enriched in a metabolite list than would be expected by chance. Pathway enrichment analysis was performed using MetaboanalystR 3.0 [43]. Comma-separated value (CSV) data files containing samples, disease status, compound IDs, and values of metabolites for each sample were generated. For compound matching, the Human Metabolome Database (HMDB) IDs provided by Metabolon were used. The auto-normalization option was selected for data normalization, which scales the data to mean = 0 and SD = 1. The quantitative enrichment analysis (QEA.) option was used, which uses the metabolite concentrations for the analysis instead of the list of differentially expressed metabolites. QEA. uses a generalized linear model to estimate a statistic (called Q-stat) of a metabolite set that describes the correlation between compound concentration profiles and phenotype [44]. This approach identifies metabolite sets when only concentrations of a few compounds are significantly different or when many related compounds have correlated small changes. The enrichment ratio (ER) is defined as the ratio of the Q-stat for the given data to its expected value by chance. ERs greater than 1 mean that the given metabolite set has different metabolite concentrations than what is expected by chance. Pathway maps were generated for visualization using the online tool Pathview (https://pathview.uncc.edu/; accessed on 23 November 2021). Pathview annotated the KEGG pathway maps [45] with normalized metabolite concentrations using red, yellow, and green to represent lower, same, and higher concentrations in CHD patients compared with controls, respectively.

### 4.5. Multivariate Analysis and Predictive Modeling

A random forest model was developed using the *randomforest* R package [46] to assess the predictive performance of inferring CHD with metabolomics data. RF models work well with relatively few samples, capture nonlinear interactions, and generalize well. The samples were divided into a training set (randomly selected 75% CHD patients (N = 735) and 75% controls (N = 2270)) and a testing set (remaining sample; N = 948). The variable importance, which is the mean decrease in accuracy if the variable is removed from the model, was calculated using the *caret* R package [47]. AUCs were calculated using the *ROCR* package in R [48]. The knee point of the variable importance curve was determined by selecting a minimum number of variables having area under the ROC comparable with the model using all metabolites. Performance of the RF model was tested in females and males separately by splitting the test set into males (N = 444) and females (N = 504). Two RF models were built: (A) based on all the metabolites (i.e., 641) and (B) based on a small subset of metabolites having the highest variable importance in model (A). Multivariate analysis was performed through least absolute shrinkage and selection operator (LASSO) using the *glmnet* R package [49]. We fit a model with 200-fold cross-validation and incorporated all 614 metabolites as well as age, sex, and BMI. The penalty parameter λ was determined based on the lowest mean error obtained by cross-validation. Two metabolite risk scores (MRS) were developed, one using all metabolites with a non-zero LASSO coefficient and a reduced model that used the metabolites with the highest LASSO coefficients. 

## 5. Conclusions

Our study has focused on a cohort from an underrepresented population, with subjects representing the Middle East and Gulf region. Similar studies should be carried on new cohorts to replicate our findings and strengthen our conclusions. Overall, our study underscores the value of metabolomics in exploring biomarkers and biological mechanisms to identify potential therapeutic targets for treating CHD.

## Figures and Tables

**Table 1:** The 45 most significant enriched pathways with FDR corrected *p* < 10^−300^.

Rank	Pathway	C	H	ER	Rank	Pathway	C	H	ER
1	Malate-Aspartate Shuttle	10	3	937.76	24	Oxidation of Branched Chain Fatty Acids	26	4	429.34
2	D-Arginine and D-Ornithine Metabolism	11	3	760	25	Mitochondrial Electron Transport Chain	19	4	422.54
3	Glucose-Alanine Cycle	13	5	740.04	26	Propanoate Metabolism	42	5	419.62
4	Gluconeogenesis	35	6	730.95	27	Tryptophan Metabolism	60	12	386.48
5	Warburg Effect	58	12	695.11	28	Ammonia Recycling	32	12	386.28
6	Citric Acid Cycle	32	7	683.41	29	Glycine and Serine Metabolism	59	21	379.37
7	Glutathione Metabolism	21	6	659.62	30	Nicotinate and Nicotinamide Metabolism	37	9	363.32
8	Lysine Degradation	30	4	638.04	31	Carnitine Synthesis	22	8	346.34
9	Pyruvate Metabolism	48	5	620.85	32	Betaine Metabolism	21	6	342.96
10	Tyrosine Metabolism	72	9	608.32	33	Sphingolipid Metabolism	40	10	341.81
11	Urea Cycle	29	12	578.21	34	Valine, Leucine and Isoleucine Degradation	60	8	328.06
12	Transfer of Acetyl Groups into Mitochondria	22	5	573.51	35	Histidine Metabolism	43	10	305.92
13	Glycolysis	25	4	572.2	36	Arachidonic Acid Metabolism	69	4	288.72
14	Phytanic Acid Peroxisomal Oxidation	26	2	571.65	37	Starch and Sucrose Metabolism	31	5	265.43
15	Phenylalanine and Tyrosine Metabolism	28	7	544.89	38	Steroidogenesis	43	3	261.33
16	Alanine Metabolism	17	7	531.58	39	Methionine Metabolism	43	15	243.09
17	Cysteine Metabolism	26	6	514.23	40	Glycerolipid Metabolism	25	6	240.15
18	Amino Sugar Metabolism	33	6	459.18	41	Pyrimidine Metabolism	59	9	155.08
19	Glutamate Metabolism	49	12	457.04	42	Fatty Acid Biosynthesis	35	8	133.24
20	Arginine and Proline Metabolism	53	16	441.27	43	Bile Acid Biosynthesis	65	11	107.6
21	Beta-Alanine Metabolism	34	8	441.12	44	Galactose Metabolism	38	7	133.77
22	Aspartate Metabolism	35	12	438.07	45	Phosphatidylcholine Biosynthesis	14	4	359.58
23	Purine Metabolism	74	12	433.8					

Rank: Rank of pathways based on descending ER; ER: Enrichment ratio; C: The number of compounds within each pathway; H: the number of tested metabolites that overlap with the pathway.

**Table 2:** The cohort characteristics after the quality control steps.

	CHD			Controls			*p* *
	Females	Males	All	Females	Males	All	
Participants, N (%)	600 (61.9)	370 (38.1)	970 (100)	1505 (50.5)	1478 (49.6)	2983 (100)	7.9 × 10^−10^
Age, Mean (SD) years	53.4 (14.5)	51.6 (14.9)	52.7 (14.6)	39.6 (11.4)	40.1 (12.6)	39.8 (12.0)	<2.2 × 10^−16^
BMI, Mean (SD) kg.m^−2^	29.8 (5.0)	31.8 (6.2)	30.5 (5.5)	28.6 (5.5)	29.4 (6.3)	29.0 (5.9)	2.7 × 10^−13^
Type 2 Diabetes ^#^, N (%)	302 (62.8)	179 (37.2)	481 (100)	135 (49.6)	137 (50.4)	272 (100)	5.9 × 10^−4^

* *p*: *p*-values were calculated using a chi-square test to compare the numbers of cases and controls with respect to sex and Type 2 diabetes. Two-sample *t*-test was used to compare age and BMI between the cases and controls. ^#^ Type 2 diabetes (T2D) status within CHD was defined as fasting blood glucose ≥ 126 mg/dL, random glucose ≥ 200 mg/dL, hemoglobin A1C ≥ 6.5%, or a prior diagnosis with oral hypoglycemic or insulin therapy. Within the controls, hemoglobin A1C ≥ 6.5% was used to define T2D patients.

## Data Availability

The data are not available in public repositories. They can be accessed through application to the Qatar Biobank through an established ISO-certified process by submitting a request online, subject to institutional review board approval by the Qatar Biobank. To submit a request, see https://www.qatarbiobank.org.qa/research/how-to-apply-new/.

## Supplementary Materials

The following supporting information can be downloaded at: https://www.mdpi.com/article/10.3390/metabo12060517/s1, Figure S1: Principal component analysis of samples. First two principal components are shown. Samples outside [µ ± 5SD] are labelled outliers. The dotted lines represent µ ± 5SD; Figure S2: Mean decrease in accuracy (variable importance) of the random forest model trained on all metabolites; Figure S3: Non-zero coefficients of metabolites in the LASSO model. Absolute value of the coefficients is plotted to identify knee point to select a subset of metabolites to develop metabolic risk score.
