# Supplementary material for: Untargeted Metabolomics Profiling Reveals Perturbations in Arginine-NO Metabolism in Middle Eastern Patients with Coronary Heart Disease
Source: Metabolites. 2022 Jun 3;12(6):517. doi: 10.3390/metabo12060517 (PMC9230991; doi:10.3390/metabo12060517)
Supplement: Supplementary file 1 [file metabolites-12-00517-s001.zip › metabolites-1677769-supplementary.pdf]

Supplementary Figures

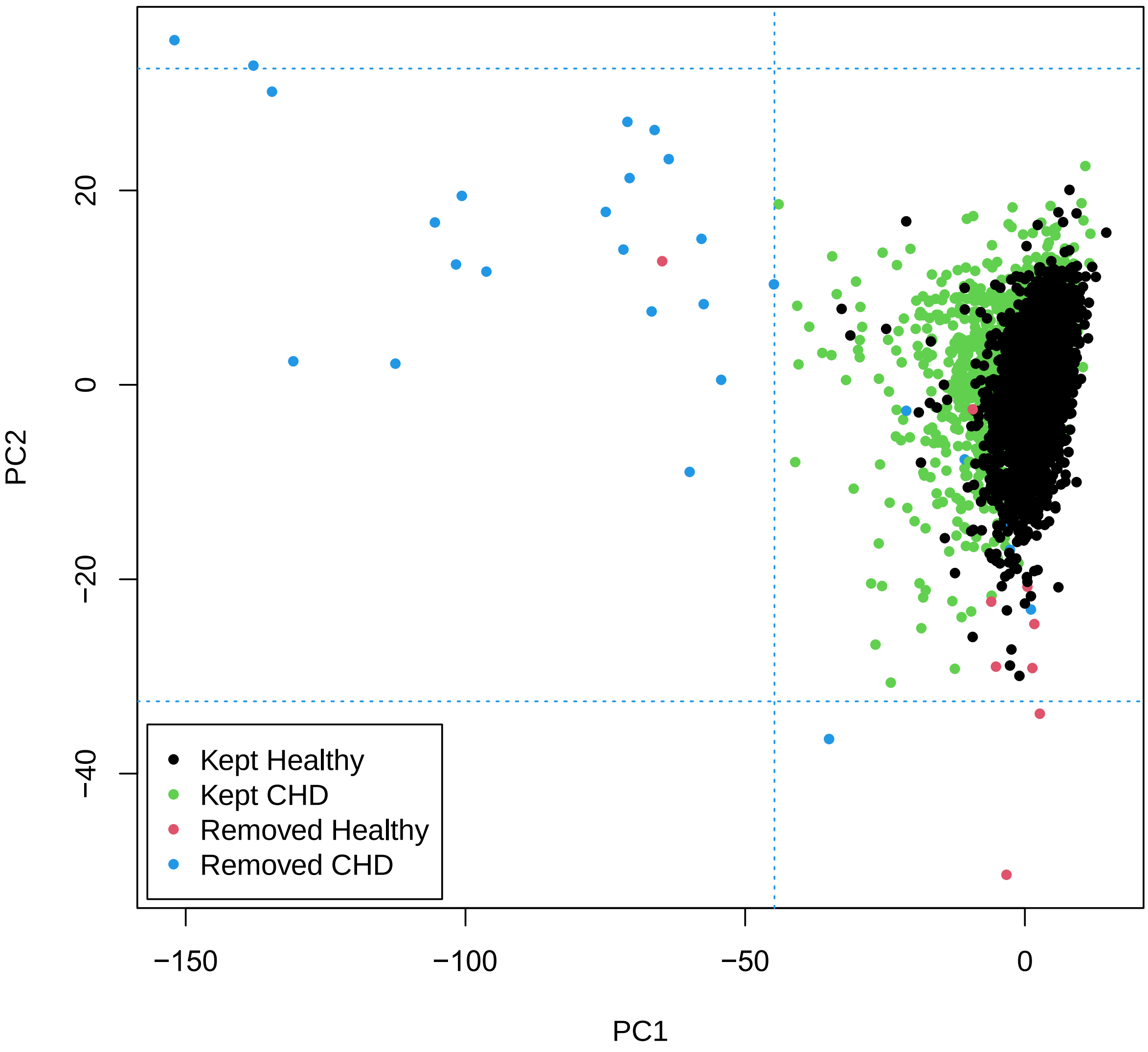

Supplementary Figure S1. Principal component analysis of samples. First two principal components are shown. Samples outside [μ ± 5SD] are labelled outliers. The dotted lines represent μ ± 5SD.

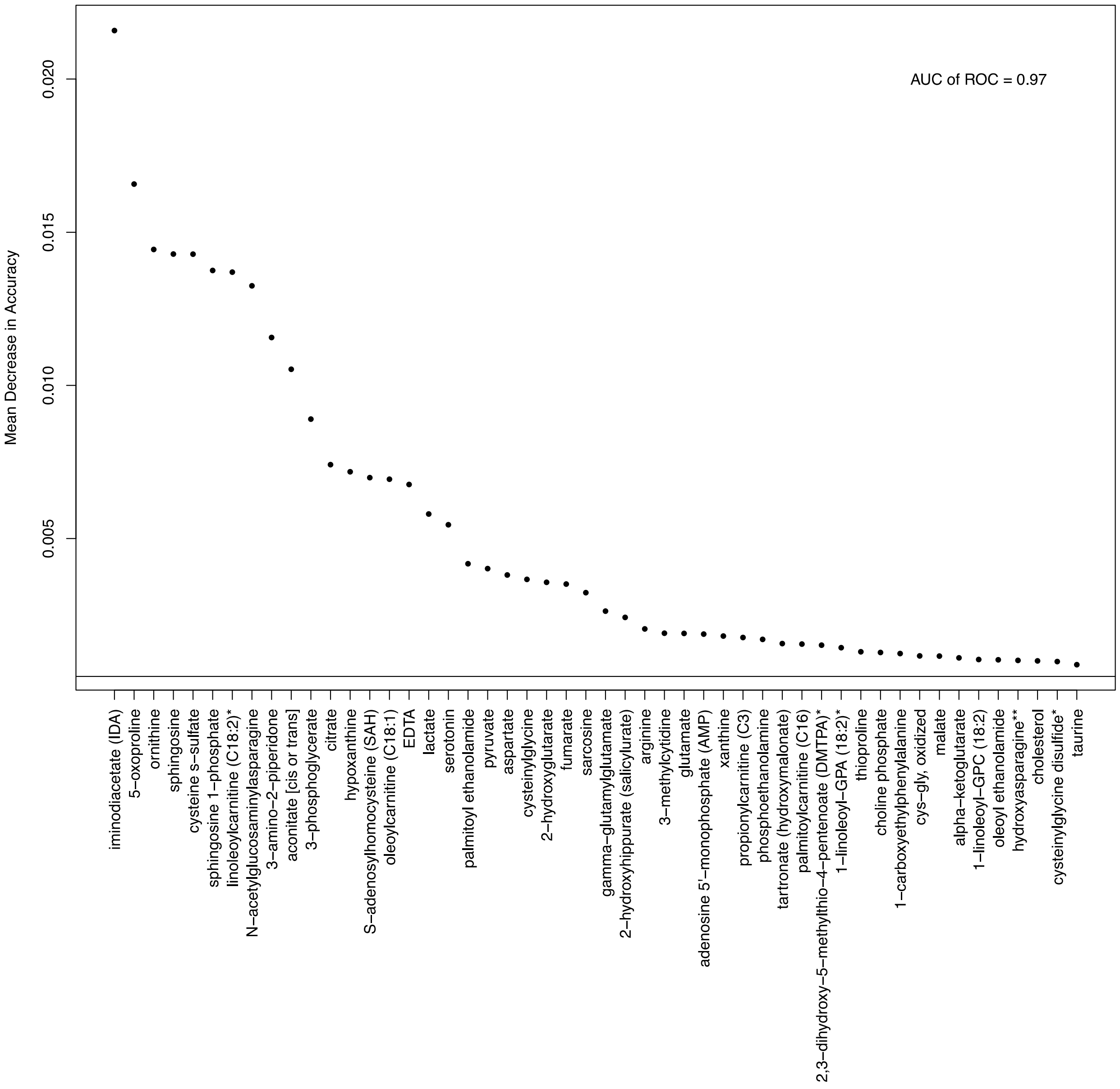

Supplementary Figure S2. Mean decrease in accuracy (variable importance) of the random forest model trained on all metabolites.

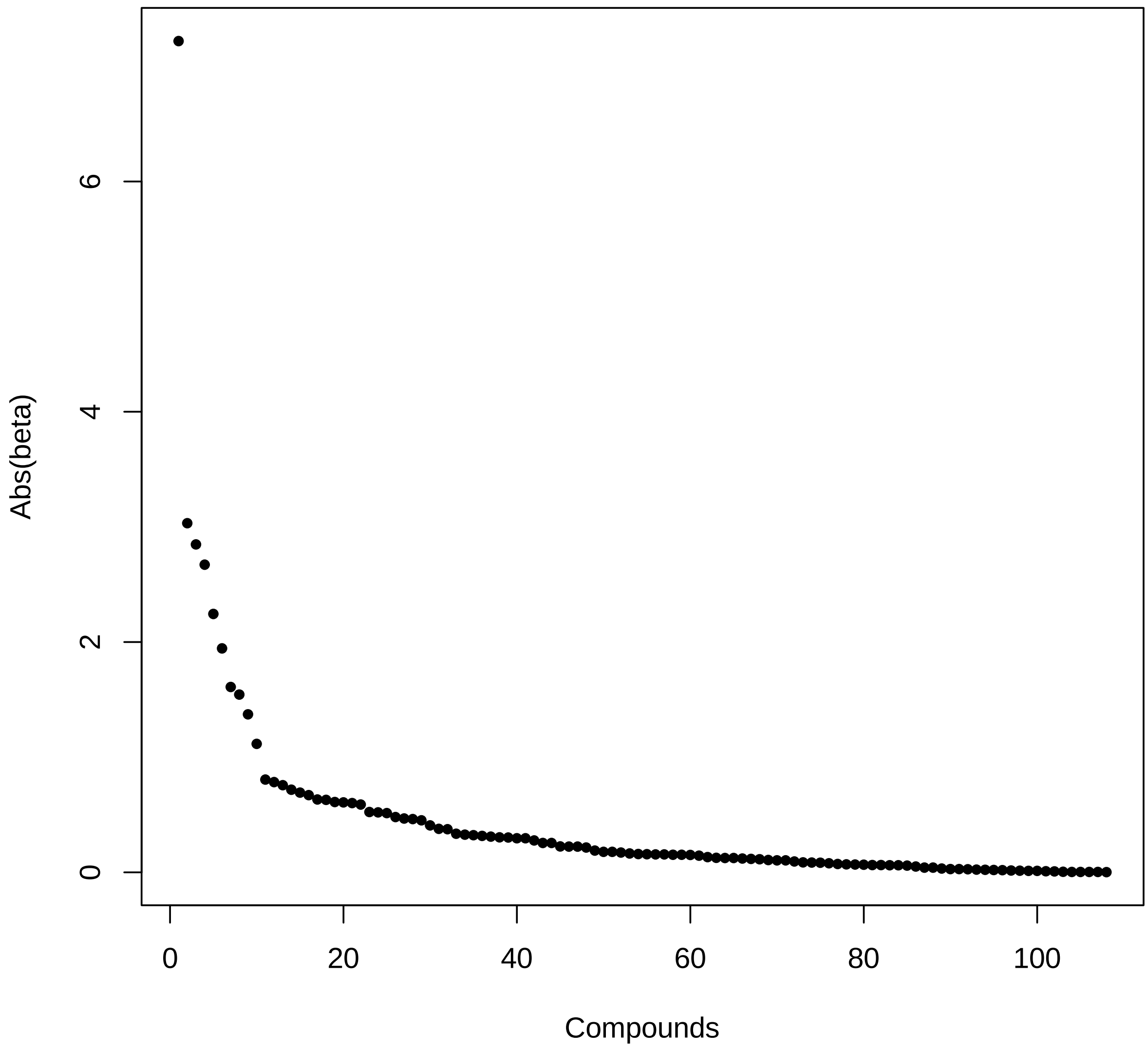

Supplementary Figure S3. Non-zero coefficients of metabolites in the LASSO model. Absolute value of the coefficients are plotted to identify knee point to select a subset of metabolites to develop metabolic risk score.
